# The Role of Radical Prostatectomy and Lymph Node Dissection in Clinically Node Positive Patients

**DOI:** 10.3389/fonc.2019.01395

**Published:** 2019-12-10

**Authors:** Giovanni Motterle, Mohamed E. Ahmed, Jack R. Andrews, R. Jeffrey Karnes

**Affiliations:** ^1^Department of Urology, Mayo Clinic, Rochester, MN, United States; ^2^Department of Surgery, Oncology and Gastroenterology–Urology, Padova, Italy

**Keywords:** cN1, lymph node dissection, prostate cancer, radical prostatectomy, staging

## Abstract

Patients diagnosed with clinically node-positive prostate cancer represent a population that has historically been thought to harbor systemic disease. Increasing evidence supports the role of local therapies in advanced disease, but few studies have focused on this particular population. In this review we discuss the limited role for conventional cross sectional imaging for accurate nodal staging and how molecular imaging, although early results are promising, is still far from widespread clinical utilization. To date, evidence regarding the role of radical prostatectomy and pelvic lymph node dissection in clinically node-positive disease comes from retrospective studies; overall surgery appears to be a reasonable option in selected patients, with improved oncological outcomes that could be attributed to both to its potential curative role in disease localized to the pelvis and to the improved staging to help guide subsequent multimodal treatment. The role of surgery in clinically node-positive disease needs higher-level evidence but meanwhile, radical prostatectomy with extended pelvic lymph-node dissection can be offered as a part of a multimodality approach with the patient.

## Introduction

In 2018 it was reported that 12–13% of PCa patients presented with regional tumor involvement at the time of diagnosis (1) and this number is likely to increase in the coming years due to novel and more accurate imaging techniques. Following the America Joint Committee on Cancer (AJCC) Staging System, the “N” refers to regional lymph nodes, namely: pelvic, hypogastric, obturator, iliac, and sacral groups. The involvement of distant LNs, namely those outside the true pelvis (for example aortic, common iliac, inguinal, supraclavicular, and retroperitoneal) is considered as M1 disease. Tumor of any “T” stage, negative for distant metastasis but with positive regional nodes involvement is referred as stage IVa (2). This considerable proportion of PCa patients has historically been treated with the assumption that the presence of lymph node metastasis indicates systemic spread of disease, thus guidelines recommend ADT as the gold standard treatment (3, 4). To date no randomized clinical trial exists evaluating the best treatment modality for these patients.

A recent systematic review (5) suggested a potential benefit in CSS and OS for patients receiving local treatment (RP or RT) for cN1 PCa vs. ADT alone. Interestingly, only one of the five studies that met the inclusion criteria included patients treated with radical prostatectomy (6), moreover most of them were redundant and population-based with connected limitations. Thus, while the overall impression is that there is a potential role for local therapy, there is still need to clarify the evidence regarding the role of surgical therapy.

In this review we aim to summarize the evidence reporting the effect of RP in cN1 population, after considering two relevant questions: can we rely on current clinical staging to exclude surgery as a possible primary treatment? Are patients with localized nodal metastasis a unique population?

## Identifying cN1 Patients

As discussed, surgery is not an option considered in current guidelines for cN1 patients, thus the correct clinical diagnosis of nodal involvement is imperative, especially aiming to minimize the rate of false positives where a potential curative intervention could be missed. However, in the following section we will review how such a strong change in treatment indications seems not supported by the performance of current N staging techniques.

### CT and MRI

Traditionally, cross sectional conventional imaging techniques such as CT or MRI are used for local and abdominal staging purposes. They assess the presence of LN involvement indirectly, by the evaluation of morphology and size. The most commonly used thresholds are short axis of nodal size of 8 mm in the pelvis and 10 mm outside the pelvis. Predictably, decreasing the threshold size increases sensitivity at the expense of specificity (7).

A meta-analysis in 2008, thus including studies mostly performed in the pre-PSA era, showed that for CT pooled sensitivity was 0.42 (95% CI 0.26–0.56) and pooled specificity was 0.82 (95% CI 0.8–0.83) (8). More contemporary studies including patients showed comparable results. Very high specificity (94–97%) of CT scan was confirmed in a cohort of 1,541 patients treated with eLND, while sensitivity was low and related to tumor characteristics, namely reaching a maximum of 24% in patients with very high risk of nodal invasion calculated with Briganti nomogram (9). The overall discrimination accuracy was low (55%). While focusing on the pN+ patients the PPV of CT scan was only 32.8%, meaning that 67.2% of patients had false positive findings on CT scans (10). Moreover the results showed that the inclusion of the information derived by CT scan did not increase the accuracy of the non-imaging-based Briganti nomogram. Similarly results were reported in another cohort of 1091 CT staged patients with a PPV of 31% (11).

MRI is increasingly used in local staging, particularly with the adoption of multi-parametric MRI in the detection and diagnosis of prostate cancer. In the aforementioned meta-analysis, MRI pooled sensitivity of 0.39 (95% CI 0.22–0.56) and pooled specificity of 0.82 (95% CI 0.79–0.83) was reported. Despite this, the differences in performance of CT and MRI were not statistically significant (8). Other studies performed with modern MRI-techniques and contemporary cohorts of patients confirmed the high specificity of MRI and suggested an improved sensitivity when DWI was incorporated in the scanning technique (12). Considerably higher sensitivity and specificity (1/0.96) were reported in a recent prospective study evaluating the performance of 3.0-T multiparametric whole body MRI in comparison with bone scan and 18F-choline PET/CT for staging purposes. However, these results should be interpreted with caution because the reference standard for true positive was derived from clinical and radiological parameters rather than from pathological evaluation (13).

### Molecular Imaging

The role of molecular imaging in the staging of recurrent prostate cancer is promising. With new radiotracers demonstrating impressive results, molecular imaging's role is likely to increase in the future (14). That being said, its role in the primary staging is still under debate and is not yet recommended in current guidelines (3, 4).

A meta-analysis of 10 studies published before 2012 and including 441 intermediate/high-risk PCa patients undergoing 18F-Choline or 11C-Choline PET/CT for nodal staging demonstrated a pooled sensitivity of 0.49 (95% CI 0.39–0.58), pooled specificity of 0.95 (95% CI 0.92–0.97), pooled positive likelihood radio of 8.35 (95% CI 4.5–15.48) and pooled negative likelihood radio 0.55 (95% CI 0.37–0.82) (15). Recently, Schiavina et al. compared the diagnostic performance of 11C-Choline PET/CT and contrast enhanced CT in a population of high risk PCa patients treated with RP +LND and reported sensitivity and specificity of 50 and 76% vs. 21 and 92%, for PET/CT and CT, respectively. Those differences were increased in those patients defined as very high-risk, suggesting this population as a potential target for PET/CT preoperative staging (16).

PSMA is a relatively new radiotracer that has recently been evaluated for promising higher sensitivity in nodal staging (17). A meta-analysis including both patients undergoing 68Ga-PSMA PET/CT for primary staging or after biochemical recurrence showed sensitivity of 0.86 (95% CI 0.37–0.98) and specificity of 0.86 (95% CI 0.03–1.00) for nodal involvement on a “per patient” basis and sensitivity and specificity of 0.8 (95% CI 0.66–0.89) and 0.92 (95% CI 0.92–0.99), respectively, on “per lesion basis” (18). Further studies evaluating the role of PMSA in the specific primary staging setting confirmed the aforementioned results (19, 20). In a prospective study, 30 patients with intermediate-high risk PCa were staged with 68Ga-PSMA PET/CT prior to surgery; on a “per patient” analysis, the sensitivity, specificity, PPV and NPV were 64, 95, 88, and 82%, respectively. On “LN-region-based” analysis, the sensitivity, specificity, PPV and NPV were 54, 99, 92, and 94%, respectively (21). Interestingly, this paper demonstrated that most missed LNs were <5 mm, consistent with one of the main limitations of PET/CT, limited spatial resolution. Considering this limitation, the marginal improvement in sensitivity and higher costs with limited availability, PET/CT is currently not recommended in primary nodal staging setting, where instead PLND remains the gold standard.

## Surgical Treatment for cN1 Patients

The rationale supporting local treatment in advanced cancers is based on the principle of tumor volume reduction and local control. The benefit of radiation therapy in addition to systemic treatment in cN1 PCa patients has been demonstrated by different studies, both in a subgroup analysis of RCT and in a population based setting (5, 22, 23). In the setting of surgery, a survival benefit of RP in patients with nodal involvement detected during surgery has led to the abandonment of frozen LN section (24, 25).

The first study to specifically analyze the role of prostatectomy in cN1 patients was reported by Moschini et al. (26); oncological outcomes of 50 (17%) cN1 M0 patients undergoing RP + PLND were compared to 252 (83%) patients with cN0, M0 disease. The authors reported no difference between groups in CSS and OS. The only significant predictors of CSM were the number of positive nodes (HR 1.10; *p* = 0.02) and pathologic Gleason score 8–10 vs. <7 (HR 2.37; *p* = 0.04) (26). Both groups were comparable in adjuvant ADT or RT. Although demonstrating promising results, the study lacked of a control group of cN1 patients treated with RT and/or ADT and such comparison is still missing.

Subsequently, a population based study from the National Cancer Database (NCBD) evaluated oncological outcomes of in 2,967 PCa patients with cN1 disease undergoing any local treatment, intended as RP or RT + ADT (*n* = 1,987) vs. ADT alone (*n* = 980). With a median follow-up of 49.7 months, in the multivariable model adjusting for selection bias, local treatment + ADT was associated with a significant overall mortality survival benefit (HR 0.31; 95% CI 0.13–0.74, *p* = 0.007). In a secondary analysis, authors compared RT + ADT and RP + ADT reporting no significant differences in overall survival between the two cohorts. Interestingly, in this population based study 67% of patients received some form of local treatment, despite of guidelines recommendation. Furthermore, 17.8% patients classified as cN1 were pN0 at RP (6).

Another population based study from SEER-Medicare database by Jang et al. evaluated oncologic outcomes between patients older than 65 years with cT3-4N1 disease, treated with RP + adjuvant RT (within 6 months after surgery) compared to RT + ADT (any ADT from 2 months before RT until 3 years after). The two cohorts were propensity matched with respect to clinical and demographic characteristics. The adjusted 10-year CSS rates for cT3N1 disease were 75.7 and 58.6% for those treated with RP + RT and RT + ADT, respectively, with a 95% CI for the difference from −0.8 to 34.2. Similarly, the 10-year OS rates were 44.3 and 40.5% for RP + RT and RT + ADT, respectively, with a 95% for the difference from −10.8 to 22.5 (27).

The aforementioned study by Seisen et al. (6) and Jang et al. (27) also compared RP and RT in cN1 patients and demonstrated contrasting results; overall these results should be interpreted that a main benefit of surgery is the prognostic information, namely the accurate staging. The importance of accurate staging in these patients can't be underestimated. Indeed, false positive rate was reported up to 20% and can be higher as already discussed in the diagnostics section. Correct staging can then guide subsequent adjuvant therapies and, in particular, can limit the adverse events related with ADT if not truly indicated. Table 1 show an overview of potential advantages and disadvantages on LND in cN1 patients.

**Table 1:** Summary of advantages and disadvantages of LND in cN1 patients.

**Advantages**	**Disadvantages**
**Surgery in cN1 patients**	
Gold standard for staging	Morbidity related to surgery
Potentially curative in limited burden	No proven oncological benefit
Cytoreduction	No clear benefit compared to RT
Avoids undertreatment Potential sequencing of adjuvant treatments	Technically challenging in advanced cases

To date no other study has directly evaluated the role of surgical treatment in cN1 patients, nor are any current clinical trials registered. Surgical treatment in this subpopulation is made of two components, namely the PLND and the RP, each of them with its own implications. The reported findings are consistent with the increasing evidence supporting local treatment for newly diagnosed metastatic PCa. As we will discuss in the following paragraph, cN1 patients represent a heterogeneous population were surgery plays a role with its cytoreductive effect (28). In particular, cytoreductive prostatectomy in metastatic patients is being evaluated in several RCTs (29, 30), in order to confirm promising results seen in retrospective series (31, 32).

The overall curative impact of LND remains controversial (33); however the interpretation of related studies may be confounded by the heterogeneous LND template employed by surgeons and the inclusion of patients with low risk of CSM. In patients with pN1 disease the removal of more LNs was reported to be associated with lower CSM rates (34) emphasizing the general recommendation of performing an extended LND in all patients with significant preoperative risk of nodal disease. The importance of treating LNs is suggested also from the studies on LND in salvage setting that, although lacking of prospective randomized data, show promising results in selected patients (35).

No definitive paradigm exists for subsequent management of pN1 patients, though early adjuvant ADT ± RT is commonly offered. A recent study demonstrated improved oncologic outcomes in patients treated with surgery and adjuvant ADT + RT compared to surgery alone or surgery and adjuvant ADT (36). These results suggest a potential role of RP as the first step in a multimodal treatment plan.

## Identifying Surgical Candidates

Most of the aforementioned studies lacked of information regarding the burden of nodal disease, thus the heterogeneity of the included patients prevented the external validity and further recommendations based on the findings.

In order to define a subgroup of patients with nodal disease who benefit the most form RP, Gandaglia et al. identified 162 patients with cN1 disease detected with conventional cross-sectional imaging and treated with RP at three tertiary centers. They reported that higher Gleason Score and higher number of clinical lymphadenopathies were the only predictors of pathological lymph node involvement in multivariate analysis. Three variables were then identified and used for stratifying patients: number of clinically positive nodes, location of nodes (intended as pelvic vs. retroperitoneal) and biopsy Grade Group. The overall 8-year CR-free and CSM-free survival rates were 59 and 80%, respectively. Differences in 8-year CR-free survival were significant for those with two or fewer lymphadenopathies vs. those with more than two (55 vs. 35%, *p* = 0.049) and in particular retroperitoneal involvement was associated with a 2-fold increase of CR (59 vs. 27% 8-year CR-free survival for pelvic only vs. retroperitoneal involvement, respectively, *p* = 0.001), other factors such as size did not have a significant impact. The multivariate model, adjusting for adjuvant or salvage therapies, showed that the site and the biopsy grade group were predictors of CR and in particular in those patients with pelvic-only LN involvement also the number of nodal stations was significant (37). This attempt to stratify patients suffers of limitations due to its retrospective nature, thus selection bias and the lack of a control group.

Despite the limitations and the need for confirmatory results, this study is interesting since it raises several discussion points. The first comes from the evidence that the size of clinical lymphadenopathies should not be used as a criterion for treatment selection; this finding seems somehow contrasting with those regarding the prognostic values of pathological LN size (38) and again emphasizes the poor accuracy of conventional cross sectional imaging. Overall, the population of cN1 patients is heterogeneous with respect to the burden of nodal involvement, comprising both those with massive lymph node involvement and those with limited nodal disease. While in the former population surgery could be interpreted as a cytoreductive treatment, in the latter it might aim to be a curative intervention. Indeed, there is evidence supporting this hypothesis reporting that patients with less than three LNs involved by PCa had better survival outcomes than those with more extended lymph node involvement, with a reported median CSS at 10-years up to 78.6% (39–41).

## M1a Disease

Non-regional lymph node metastases are classified as M1a disease (2), in particular common iliac and retroperitoneal nodes are included in this classification. As aforementioned, most of the studies evaluating both primary and salvage treatment for nodal disease suffered from the heterogeneity of the definitions used to describe nodal involvement and LND extension, which sometimes was performed up to the aortic bifurcation (thus including common iliac nodes). The basis of this considerations refers to a pathological mapping study which showed that patients with positive retroperitoneal lymph nodes always had positive lower pelvic lymph nodes, regardless of the location of the nodal area involved, and in particular the common iliac nodes were always involved, suggesting an ascending pathway of metastases starting form lower pelvic nodes to retroperitoneal chains through common iliac nodes (42). While the extension of the disease outside the true regional nodes appears as mirror of systemic disease, there is still debate whether patients classified as M1a are comparable in terms of treatment and outcomes with those with osseous and visceral metastases. In a SEER-based study performed by Culp et al. the subgroup analyses surprisingly showed improved OS in patients treated with RP for M1b (*p* < 0.001) and M1c (*p* < 0.001) disease in comparison with M1a. These results should be however interpreted cautiously because of the lack of information regarding the pelvic node dissection, in addition to the known lack of information regarding ADT typical of SEER studies (31). A study by Moschini et al. evaluated the oncological outcomes of 17 cM1a patients treated with combined pelvic and formal retroperitoneal lymph node dissection up to the renal vessels and a minimum of 6 months of ADT; they found that the CSM-free survival at 5 years was 80.2% in M1a patients compared to 49.0% in M1b but not reaching significant *p*-value (43). While often included in studies evaluating RP in metastatic PCa, stratified outcomes for M1a patients including adequate nodal dissection are missing. Recently, another SEER study test the association of baseline PSA and local treatment within different M1 substages in a propensity matched cohort; M1a patients receiving local treatment (RP or RT) had lower CSM than those not treated with local treatment (HR 0.32 95% CI 0.17–0.60, *p* < 0.001) (44). In the setting of node-only recurrent prostate cancer a prognostic model has been recently developed to predict those who benefit the most from salvage LND, namely those with real oligorecurrent disease: among the others, a number of PET/CT detected nodes > 2 (HR 1.26 95% CI 1.05–1.61, *p* = 0.019) and nodes in the presence of nodes in the retroperitoneum (HR 1.24 95% CI 1.01–1.52, *p* = 0.038) were predictors of worse outcomes (45). To date for newly diagnosed cM1a patients there is insufficient evidence supporting an additional oncological benefit of RP and LND, even though from the experiences in salvage super-extended lymph node dissection could suggest a rationale in supporting lymph node dissection up to the retroperitoneum, in particular when considering also the potential prevention of local complications derived from advanced prostatic and nodal involvement.

## Discussion

This review represents the most comprehensive and updated summary of current evidence regarding the oncologic outcomes of radical prostatectomy in cN1 patients to our knowledge. The evidence and rationale provided support an oncological benefit of RP + LND in this setting, consistently with a recent systematic review including studies mainly focused on RT (5). A quick review of the imaging techniques for N staging showed overall poor performance of conventional cross sectional imaging, this could result potential under/overtreatment of patients. The potential better performance of molecular imaging is still not considered sufficient by guidelines to be implemented in the primary staging (3, 4) and extended LND remains the gold standard for nodal staging and thus guiding subsequent treatments and follow-up.

To the best of our knowledge, all studies evaluating the role of RP in cN1 patients are retrospective and no randomized clinical trial has ever been performed or is currently recruiting cN1 patients in order to evaluate the best treatment options. Additionally most of the retrospective studies lack of adequate assessment of subsequent adjuvant therapies, especially population based ones. These limitations must be taken in consideration while reading these results and this makes the quality of the evidence insufficient for definitive recommendations.

The reported evidence is indeed including great variety of cN1 patients, namely those with false-positive imaging and pN0 disease at surgery, those with minimal nodal involvement and those with massive and sometime extra-pelvic nodal disease. While for pN0 patients the curative role of surgery is clear and treating these patients with primary ADT would result in unacceptable under-treatment, patients with limited nodal involvement could benefit from the curative intent of surgery too. Indeed, patients with pN1 disease after RP + LND without ADT showed 10 year-BRFS of 28% and patients with low nodal burden and GS <8 represented the most favorable group (41). Furthermore, the rationale of maximizing local control comes from the observation that also both surgical margins and local disease stage represent significant predictors of oncological outcomes (46). Although current classification (2) do not distinguish subcategories of N positive patients, evidence seems to support clear different oncological outcomes between those with low nodal burden of disease (two or less LNs) and those with more extended disease (37, 39–41). The latter are probably the ones in which the old assumption that nodal involvement equals to systemic disease is true, but even in this case it remains questionable whether surgery could play cytoreductive role both on the prostate (47, 48) and the lymph nodes (35).

It is not surprising that the only comparison of surgery and radiation in cN1 patients failed to show any benefit (6); there is however increasing evidence supporting the role of radiation in adjuvant setting after surgery revealing pN1 disease, especially for those patients with low nodal burden of disease and lower tumor grading (49). In light of these considerations, there is strong need of high-quality evidence regarding outcomes of surgery in this particular population; while awaiting results of potential RCTs other retrospective data could be useful too. Indeed data from population databases of the reported studies show that, even if not recommended by guidelines, surgery in cN1 patients is not uncommon in clinical practice.

## Conclusion

Treatment of clinically diagnosed nodal involvement of PCa remains controversial, with not negligible evidence supporting an oncological benefit derived from radical prostatectomy and pelvic lymph node dissection. This remains a considerable proportion of patients who are not staged properly with conventional imaging techniques and may be undertreated. There is absolute need of prospective randomized data clarifying the role of surgery and its timing in the setting of a multimodal treatment.

## Author Contributions

GM and RK: conception of the work. GM: acquisition and interpretation of data. GM, JA, and MA: drafting the manuscript. JA, MA, and RK: critical revision for important intellectual content.

### Conflict of Interest

The authors declare that the research was conducted in the absence of any commercial or financial relationships that could be construed as a potential conflict of interest.

## Abbreviations

(e)LND: (extended) pelvic lymph node dissection
ADT: androgen deprivation therapy
BRFS: biochemical recurrence free-survival
cN1: clinically node positive
CR: clinical recurrence
CSM: cancer specific mortality
CSS: cancer specific survival
LN: lymph node
OS: overall survival
PCa: prostate cancer
PPV: positive predictive value
RP: radical prostatectomy
RT: external beam radiation therapy.
